# Effect of Interface Modified by Graphene on the Mechanical and Frictional Properties of Carbon/Graphene/Carbon Composites

**DOI:** 10.3390/ma9060492

**Published:** 2016-06-20

**Authors:** Wei Yang, Ruiying Luo, Zhenhua Hou

**Affiliations:** School of Physics and Nuclear Energy Engineering, Beihang University, Beijing 100191, China; yangwei_vip@hotmail.com (W.Y.); houzhenhualove@126.com (Z.H.)

**Keywords:** brake materials, carbon, graphene, Weibull, hardness

## Abstract

In this work, we developed an interface modified by graphene to simultaneously improve the mechanical and frictional properties of carbon/graphene/carbon (C/G/C) composite. Results indicated that the C/G/C composite exhibits remarkably improved interfacial bonding mode, static and dynamic mechanical performance, thermal conductivity, and frictional properties in comparison with those of the C/C composite. The weight contents of carbon fibers, graphene and pyrolytic carbon are 31.6, 0.3 and 68.1 wt %, respectively. The matrix of the C/G/C composite was mainly composed of rough laminar (RL) pyrocarbon. The average hardness by nanoindentation of the C/G/C and C/C composite matrices were 0.473 and 0.751 GPa, respectively. The flexural strength (three point bending), interlaminar shear strength (ILSS), interfacial debonding strength (IDS), internal friction and storage modulus of the C/C composite were 106, 10.3, 7.6, 0.038 and 12.7 GPa, respectively. Those properties of the C/G/C composite increased by 76.4%, 44.6%, 168.4% and 22.8%, respectively, and their internal friction decreased by 42.1% in comparison with those of the C/C composite. Owing to the lower hardness of the matrix, improved fiber/matrix interface bonding strength, and self-lubricating properties of graphene, a complete friction film was easily formed on the friction surface of the modified composite. Compared with the C/C composite, the C/G/C composite exhibited stable friction coefficients and lower wear losses at simulating air-plane normal landing (NL) and rejected take-off (RTO). The method appears to be a competitive approach to improve the mechanical and frictional properties of C/C composites simultaneously.

## 1. Introduction

Carbon/carbon (C/C) composites have been widely used as structural and braking materials in the aerospace industry [1,2,3,4,5]. As the aviation industry continues to develop, current structural and braking materials are subjected to increasing speeds and larger dynamic loads during their application. Thus, more excellent properties of C/C composites are required. The mechanical and frictional properties of these materials, which are strongly determined by the microstructure and properties of the carbon fibers, carbon matrix, and fiber/matrix interface [6], should be improved. The performance of C/C composites can be improved by surface treatment of carbon fibers and matrix modification.

Graphene, a recently discovered carbon material, possesses a two-dimensional structure and exhibits extraordinary mechanical [7], electrical [8], and thermal [9,10] properties. Graphene presents self-lubrication properties and good wear resistance. Unlike in carbon nanotubes and nanofibers, modification only improves the mechanical properties of braking composites. In addition, braking composites suffer from friction coefficient instabilities [11,12,13], which can cause their wear rate to increase. While numerous works have focused on the mechanical and frictional properties of graphene/polymer and graphene/metal composites [14,15,16], few studies describing C/C composites are available.

In the work reported here, we take advantage of the excellent weaving properties of polyacrylonitrile-based carbon fibers (PANCFs) to improve the preform of C/C composites [17,18]. We developed a simple and effective strategy to manufacture a graphene-modified C/C (C/G/C) composite and investigated the mechanism of direct graphene grafting onto the carbon fibers. The effects of modification on the microstructural, mechanical, thermal and frictional properties of the resulting C/G/C composite were then investigated.

## 2. Material and Methods

### 2.1. Materials

Polyacrylonitrile carbon fiber (12k PANCF) (Tuozhan Company, Yantai, China) preforms were constructed from layers (0° and 90°) of non-woven long fiber cloth and short-cut fiber web and then needle-punched. The preforms showed a density of approximately 0.55 g/cm^3^. The size of the preforms was Φ = 100 mm × 15 mm.

### 2.2. Preparation of Samples

#### 2.2.1. Preparation of Graphene Oxide

Graphene oxide (GO) was synthesized according to the literature methods [19,20]. In a typical procedure, 3 g of graphite (flake size about: 1–5 μm), 3 g of NaNO_3_, and 200 mL of H_2_SO_4_ were mixed together in an ice bath for 30 min. Then, 12 g of KMnO_4_ was slowly added into the mixture while stirring, and the rate of addition was controlled in the ice bath. The mixture was then transferred to a 30 °C water bath and stirred for about 30 min. Subsequently, 200 mL deionized water was added dropwise into the mixture with stirring. After 15 min, the mixture was further treated with 700 mL of deionized water and 60 mL of 30% H_2_O_2_ solution. The solution was then filtered and subjected to wash with deionized water until the pH value of reached 6. GO suspension was collected in a seal vessel.

#### 2.2.2. Preparation of C/G/C Composites

**Figure 1:**
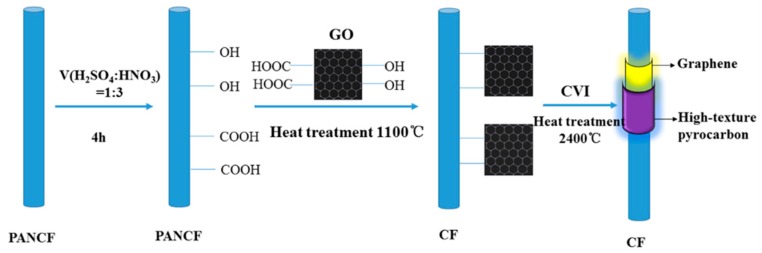
Schematic of C/G/C composite preparation.

C/G/C composites were fabricated as follows in Figure 1: (1) Preforms were oxidized by a mixture of 3:1 (*v*/*v*) concentrated H_2_SO_4_ and HNO_3_ and then sonicated for 4 h at 60 °C. Then, the carbon fibers were taken out and washed several times with deionized water until the pH of the wash water was neutral and then dried under vacuum; (2) The GO (1 g) was placed in a round-bottomed flask (500 mL) and dispersed in deionized water for 1 h; (3) The preforms were placed in the round-bottomed flask for 12 h at 25 °C under sonication and then dried at 80 °C under vacuum; (4) The preforms were heat-treated at 1100 °C for 2 h; (5) After grafting of the graphene sheets on the surface of the carbon fibers, the preforms were densified using natural gas (mainly contain CH_4_) and C_3_H_8_ through CVI at 1080–1100 °C with total pressure of 1–5 kPa. The mass ratio of natural gas:C_3_H_8_ was 2:1 [21]. Preforms without graphene were densified in the same manner for comparison with the C/G/C composite. After final graphitization at 2400 °C for 2 h, the final density of both composites was found to be 1.72 ± 0.02 g/cm^3^.

### 2.3. Characterization

The microstructure of the composites was characterized through polarized light microscopy (PLM, 2700P, Leica, Solms, Germany) and scanning electron microscopy (SEM, Hitachi SU8000, Tokyo, Japan). The polished surfaces of the C/C and C/G/C composites were analyzed by Raman spectrometry (LabRAM, HR800, Jobin Yvon, Paris, France) at a laser excitation wavelength of 514.5 nm. The dynamic properties of the specimens were determined using dynamic mechanical analysis (DMTA, TA Instruments 2980, New Castle, DE, USA) through three point bending forced vibration tests in air from 50 to 450 °C. The specimens for the DMTA test had the nominal dimensions of 2 mm × 6 mm × 60 mm. The specimens all cut from the fabricated composites. In this test, the span was 40 mm, and the loading direction was perpendicular to the cloth layer direction. The heating rate was 5 °C/min. According to ASTM E756-05 (2010) [22], the internal friction (*Q*^−1^) was measured according to Equation (1) as follows:

(1) $$Q^{-1}=\tan\delta =\frac{E^{\prime \prime }}{E^{\prime }}$$

where δ is the loss angle between the applied stress and strain, *E*′ is the dynamic storage modulus, and *E*″ is the loss modulus.

The flexural properties of composites were tested using a three point bending test at room temperature (RT), 0.5 mm/min loading speed, and 60 mm support span. The specimens were formed as rectangular bars with dimensions of 6 mm × 10 mm × 80 mm. Flexural strength (σ*_f_*) was calculated using the following Equation (2) [23]:

(2) $$\sigma_{f}=\frac{3PL}{2bh^{2}}$$

where *L* is the span of the bend test, (mm); *h* is the thickness of the specimen, (mm); *b* is the width of the specimen, (mm); and *P* is the maximum load.

Interlaminar shear strength (ILSS), τ*_s_*, was calculated using the following Equation (3) [23]:

(3) $$\tau_{s}=\frac{3P}{4bh}$$

**Figure 2:**
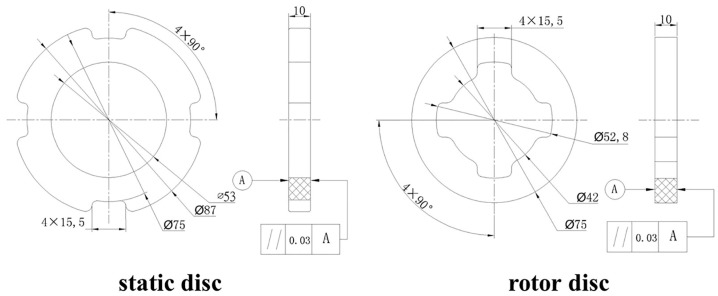
Schematics of the composites brake discs (unit: mm).

The braking performance of both composites during simulated airplane normal landing (NL) and rejected take-off (RTO) The test standard is Test methods for aircraft wheel friction materials (HB 5434.7-2004 [24]). The property of friction was measured using a wear system (MM-2000, Xian Test Factory, Xi’an, China). There were using a friction tester in air with a disc-on-disc configuration. The size of rotor and static discs were shown In Figure 2. Those discs were machined from the same composites. The braking parameters for the braking test were obtained (Table 1). All braking experiments were performed under dry conditions. Under the same braking conditions, each test was repeated 10 times. Mass wear was defined as the mass loss of both discs (rotor and static). Mass wear measured by an analytical balance after 10 braking cycles. Temperature was measured using a thermocouple. The thermocouple position located 1 mm behind the worn surface of the static disc.

The average friction coefficient of each braking test was calculated as the following Equation (4):

(4) $$\mu =2M/\left[BS(r_{1}+r_{2})\right]$$

where *M* is average moment (N·m), *B* is average brake pressure (MPa), *S* is worn surface area (m^2^), *r*_1_ is the average friction radius of the rotor, and *r*_2_ is the average friction radius of the static disk (m).

**Figure 3:**
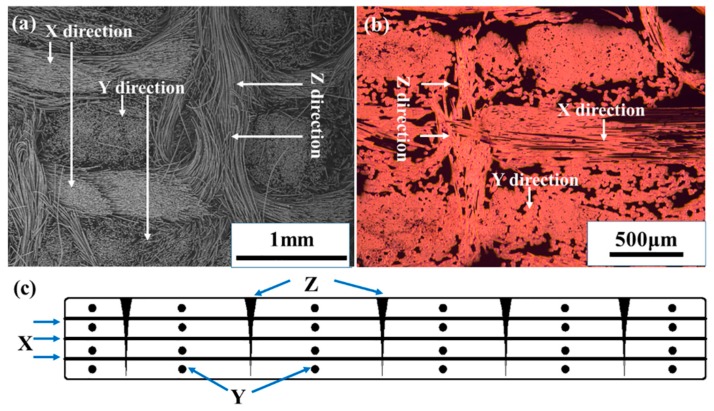
The direction of fiber in brake discs: (**a**) untreated preforms; (**b**) carbon/carbon composites; (**c**) schematic cross section.

In Figure 3, the *X*, *Y* and *Z* directions are clearly shown. The preform is constructed from layers (0°, *X* direction) and layers (90°, *Y* direction) of non-woven long fiber cloth and needle-punched fibers (*Z* direction) (Figure 3a). In addition, we can also find *X*, *Y* and *Z* directions after densification in Figure 3b. The cross section of carbon direction model diagram is exhibited in Figure 3c.

Thermal conductivity was measured using a laser-flash system (LFA 457, NETZSCH, Munich, Germany). The sample is Φ10 mm × 2 mm. According to ASTM C1783-15 [25], the thermal conductivities of C/C and C/G/C composites were calculated as the following Equation (5):

(5) $$\alpha =c_{p}\cdot \lambda \cdot \rho$$

where α is the thermal conductivity (W·m^−1^·K^−1^), λ is the thermal diffusivity (m^2^·s^−1^), *c*_p_ is the specific heat (J·kg^−1^·K^−1^), and ρ is the density (kg·m^−3^) of the composite.

The hardness of pyrocarbon matrices was determined on polished surfaces by nanoindentation measurements (MTS Nanoinstruments, Knoxville, TN, USA). The Nanoindenter XP system test with a Berkovich-type pyramid-shaped diamond indenter. The indenting load on the fibers was applied parallel to the fiber axis. Nanoindentation hardness was determined from the load–displacement (*F*–*h*) curve. The indentation load (*F*) and displacement (*h*) were continuously recorded during one complete loading and unloading cycle. Hardness, *H*, was calculated using the following Equation (6) [26,27]:

(6) $$H=\frac{F_{\max}}{A}$$

where *F*_max_ is the maximum load, *A* is the projected area of contact, which could be deduced from an empirically determined function described by Equation (7):

(7) $$A=\alpha {\left(h_{\max}-\varepsilon \frac{F_{\max}}{S_{\max}}\right)}^{2}$$

where α and ε are the apical angles of the indenter. For the Berkovich indenter, these parameters are assigned values of 26.44 and 0.75, respectively. *S*_max_ is the initial unloading stiffness, *S* = d*F*/d*h*.

For the single fiber/matrix push-out test, samples were sliced and then polished to a final thickness of approximately 40–100 μm. Before testing, the surface of each specimen was inspected by the nanoindenter’s light microscope to select the fibers with perpendicular orientation. Fiber push-out tests were carried out with a constant displacement rate by Nano Indenter XP system with a 60° cone indenter with 5 μm diameter flat-end tip. The specimen was placed on a specimen holder with a groove of 40 μm in width. Fibers in composites above the groove were loaded by triangular diamond pyramidal indenter, where the maximum load was 108 mN. About 20 times push-out were conducted for each specimen. The interfacial debonding strength (IDS) was defined by Equation (8) [28]:

(8) τ=P/πDt

where *P* is the push-out load, and *D* and *t* are the fiber diameter and specimen thickness, respectively.

Thermal gravimetric analyses (TGA, STA 449C, NETZSCH, Munich, Germany) were used to evaluated the content of the grafted GO, with a heating rate of 10 °C·min^−1^ in an argon atmosphere.

## 3. Results and Discussion

### 3.1. Morphology and Microstructure

**Figure 4:**
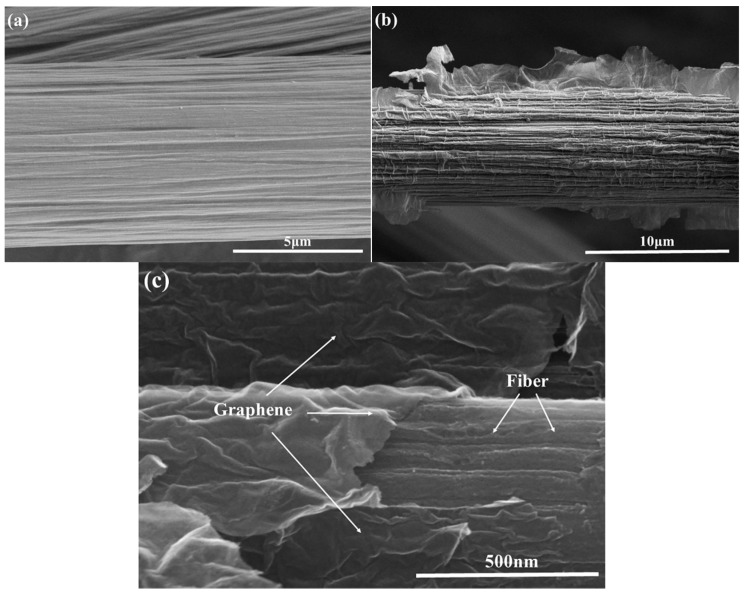
SEM images of: (**a**) untreated carbon fibers; (**b**) graphene-modified carbon fibers and (**c**) high-magnification for graphene on the fiber.

The surface topography of the fibers was characterized by SEM. Figure 4 shows SEM images of the untreated and graphene-modified carbon fibers. Marked differences in the surface topographies of the fibers are evident. In Figure 4a, the untreated fibers appear to be relatively neat and smooth. Several narrow grooves are distributed parallel to the longitudinal direction of the CF [29]. After graphene is grafted, we can find in Figure 4b,c, graphene is grafted onto the fiber surface, forming a new inflationary structure with increased carbon fiber surface roughness and provided nuclei for pyrocarbon during chemical vapor infiltration. These characteristics could significantly increase interfacial bonding strength by increasing the fiber surface area and enhancing mechanical interlocking between the fibers and the carbon matrix [30,31].

**Figure 5:**
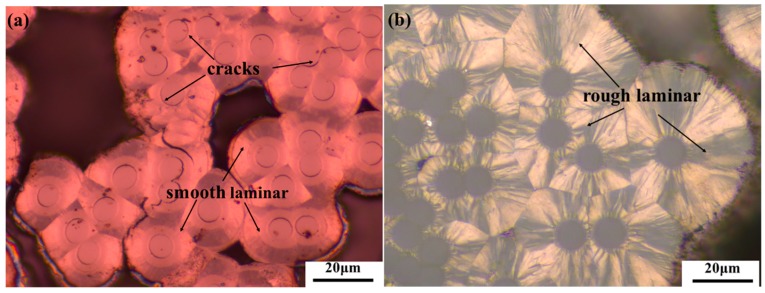
Polarized light microscopy images of the (**a**) C/C and (**b**) C/G/C composites.

Figure 5 shows two optical light micrographs of the fabricated composites. In Figure 5a, the matrix of the C/C composite is mainly composed of smooth laminar (SL) pyrocarbon, which shows numerous ring cracks between the fiber and the matrix [32]. By comparison, due to the presence of graphene, the matrix of the C/G/C composite is mainly composed of rough laminar (RL) pyrocarbon in Figure 5b [33,34]. The large surface energy of graphene may induce the formation of a highly textured pyrocarbon during chemical vapor infiltration (CVI). Due to the π–π conjugated electronic structure in graphene, graphene can act as nuclei of hydrocarbon molecules. Specifically, the sp^2^ hybridization formed big π orbitals attract similar structured small polyaromatic molecules by Van Der Waals forces in the direction that vertical to the surface of graphene. That is to say, graphene on the surface of carbon fibers can induce the ordered deposition of PyC (high texture carbon matrix) around carbon fibers during CVI [35,36,37].

**Figure 6:**
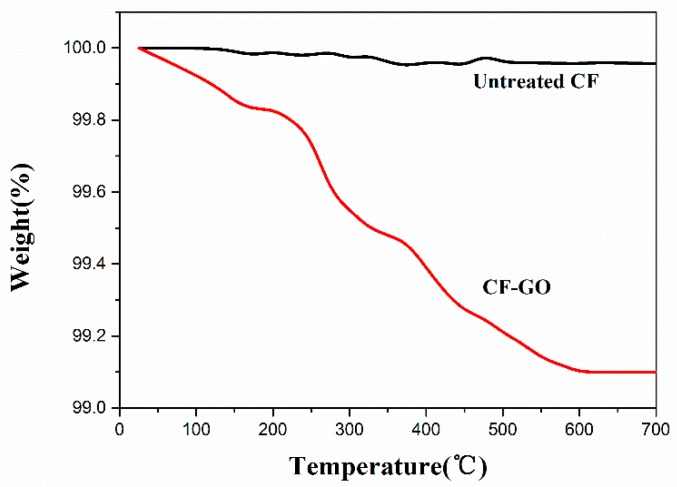
TGA curves of untreated and grafted carbon fiber.

In order to obtain the content of GO, TGA measurements were carried out. Figure 6 presents the TGA results of the untreated carbon fiber and the CF-GO. Typically, the weight loss (about 0.9%) between 25 and 600 °C was mainly attributed to the decomposition of the oxygen-containing groups. The ratio of GO to CF in treated carbon fiber can be assessed via the TGA spectra values. The weight content of GO and fibers in the preform is 0.9 and 99.1 wt %, respectively. After densification, the C/G/C composites have been treated at 2400 °C for 2 h. The density of preform and C/G/C composites are 0.55 and 1.72 g/cm^3^, respectively. Therefore, the content of carbon fibers, graphene oxide and pyrolytic carbon can be calculated by weight increase. Finally, the weight contents of carbon fibers, graphene and pyrolytic carbon are 31.6, 0.3 and 68.1 wt %, respectively.

**Figure 7:**
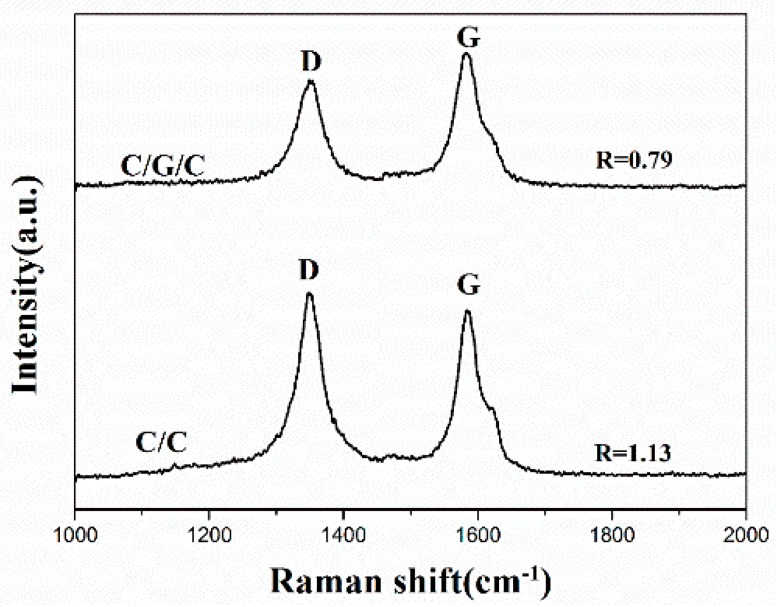
Raman spectra of the C/C and C/G/C composites.

Raman signal is very sensitive to the disorders contained in materials. In Figure 7, both composites exhibit distinct two prominent peaks (approximately 1350 and 1580 cm^−1^), which correspond to the D band assigned to defects within the carbon lattice and G band of the symmetry vibration mode for graphite, respectively. The lower intensity ratio *R* (*I*_D_/*I*_G_) of the D peak and G peak corresponds to the higher quality of graphitization within carbon materials [38,39]. While the *R* of the SL matrix of the C/C composites is 1.13, the *R* of the RL matrix of the C/G/C composites is 0.79. The C/C and C/G/C composites considerably differ in R values, indicating that the graphene considerably affects the microstructure of pyrolytic carbon near the fiber surface and also induce high-textured carbon matrix. It is clear that the nuclei effect of graphene for the high-texture pyrolytic carbon formation is much more obvious than the C/C composites without graphene.

**Figure 8:**
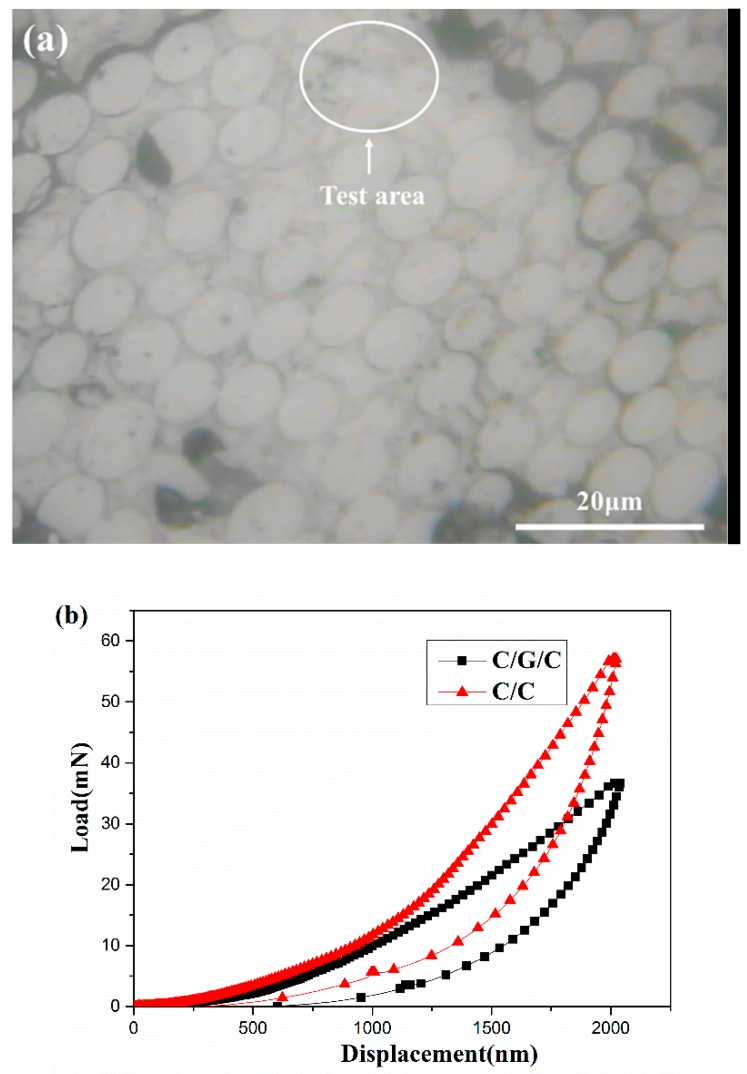
The nanoindentation experiments: (**a**) the microscope image of sample surface; and (**b**) typical load–displacement curves of nanoindentation of the composites.

The surface of each specimen was inspected by the nanoindenter’s light microscope to select the carbon matrix in Figure 8a. Typical load–displacement curves of the matrices of both composites are shown in Figure 8b. The maximum load at 2000 nm displacement of the C/C composite is higher than that of the C/G/C composite. The numbers indicated in the figure are the mean values of five measurements. The average hardness values of the matrices of the C/G/C and C/C composites are 0.473 and 0.751 GPa, respectively. Although these data do not represent the real hardness of the composites at braking, the data can be used to compare the matrix of C/G/C and C/C composites. The low hardness of the C/G/C composite matrix is mainly due to its fabrication process. The surface of the fibers modified by graphene can produce more active sites and achieve a highly textured pyrocarbon.

To study the effect of the interface modified by graphene on interfacial debonding strength, single fiber push-out tests of C/C composites and C/G/C composites were carried out. The push-out phenomenon occurs may preliminarily be interpreted as the push-out movement of a enough thin specimen, when the push-out stress conquers the shear stress between fiber and matrix [27,40]. In this study, the push-out load for specimens obtained from the test was used to calculate corresponding interfacial shear stress of the composites. The average interfacial debonding strength of C/C composites and C/G/C composites were 7.6 and 20.4 MPa, respectively. Thus, the C/G/C composites generally show higher interfacial debonding strength than the C/C composites. These results indicated that the interfacial debonding strength of composites was significantly improved by the presence of the graphene layer because this layer strengthens the interface bonding state between fiber and matrix of the composites.

### 3.2. Static Mechanical Performance

Bending tests were conducted on 20 samples of each composite. Preforms were subjected to Weibull analysis of their flexural strength, and two parameters, σ_0_ and m were determined by Equation (9):

(9) $$\ln\text{​}\ln\left(\frac{1}{1-P(\sigma_{i})}\right)=m\text{​}\ln\sigma_{i}-m\text{​}\ln\sigma_{o}$$

**Figure 9:**
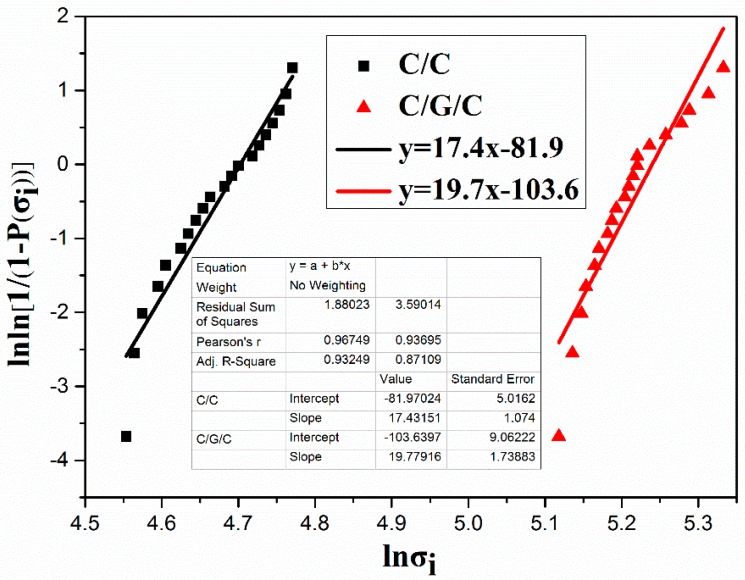
Weibull plots of the flexural strength of the C/C and C/G/C composites.

Figure 9 plots ln[1/(1 − *P*(σ*_i_*))] *versus* ln(σ*_i_*); here, m represents the slope of the fitted straight line and σ_0_ is the flexural strength of the composites. The analysis results of the Weibull parameters (*m* and σ_0_) are summarized in Figure 9. The Weibull shape parameter, which is another important factor [41] that can considerably influence the performance of the final carbon fiber composites, increases from 17.4 in the C/C composite to 19.7 in the C/G/C composite. These results are in agreement with those of other researchers, who showed that the grafting process is advantageous to flexural strength [42,43,44,45]. The average flexural strengths of both composites are approximately equal to the scale parameter of the distribution (σ_0_) [46,47]. The σ_0_ values of the C/C and C/G/C composites are 110 and 188 MPa, respectively. The average flexural strength of the C/G/C composite is 187 ± 12 MPa, much higher than that of the C/C composite (106 ± 10 MPa). Compared with untreated carbon fiber, the flexural strength of functionalized carbon fiber composites was increased by 76.4%, which was higher than that of previous studies [48,49,50].

**Figure 10:**
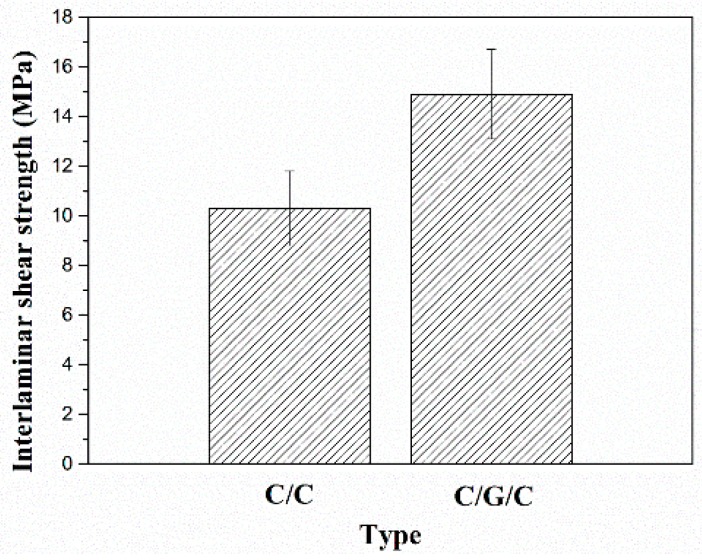
Interlaminar shear strength of the C/C and C/G/C composites.

ILSS tests were conducted on 6 samples of each composite. The ILSS results of C/C and C/G/C composites are shown in Figure 10. Evidently, graphene grafting significantly increases the ILSS of the composites. The ILSS value of the C/G/C composite is 14.9 ± 1.8 MPa, much higher than that of the C/C composite (10.3 ± 1.5 MPa). From results of flexural strengths, IDS and ILSS, we can find that the mechanical properties of the composites are greatly affected by fiber/matrix interface.

### 3.3. Dynamic Mechanical Properties

**Figure 11:**
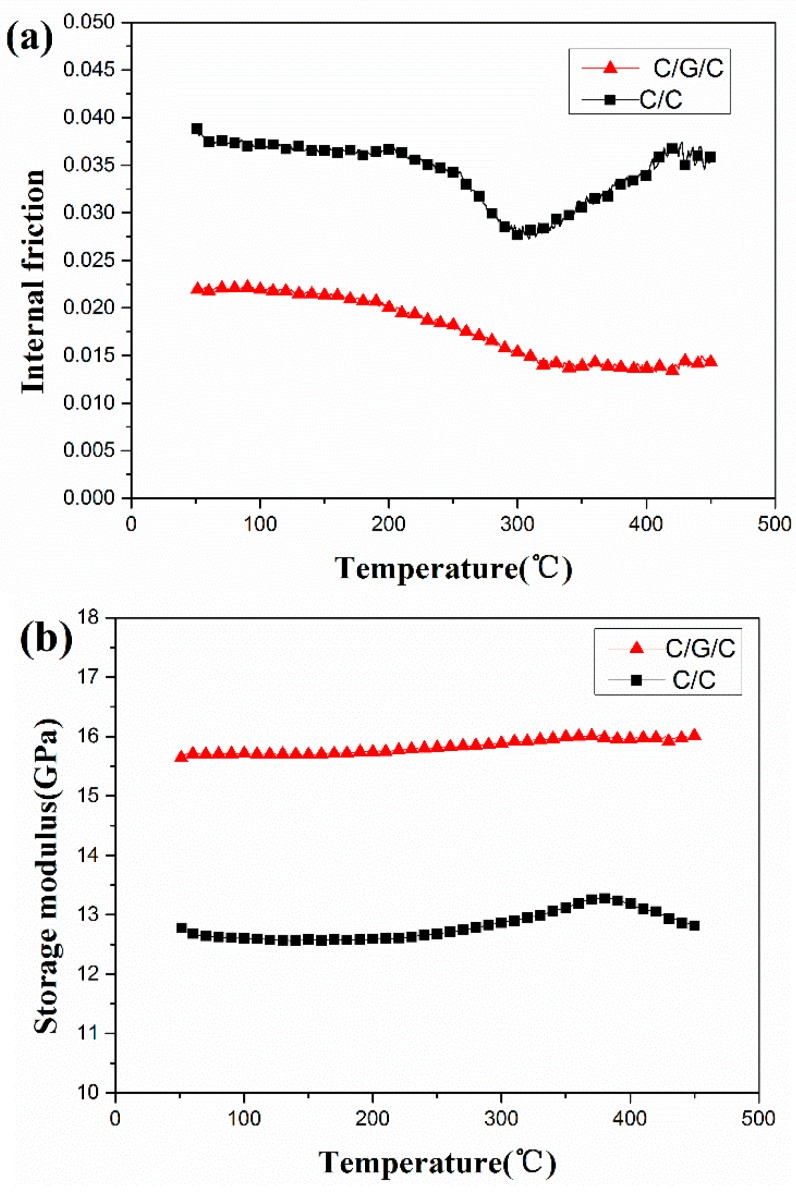
Dynamic mechanical properties of the composites *versus* temperature at 10 Hz and 0.025% strain: (**a**) internal friction; and (**b**) storage modulus.

In Figure 11, the internal friction and storage modulus of both composites depends on the testing temperature. As temperature increases from 50 to 450 °C, the internal friction of the C/C composite decreases gradually and then begins to increase, as shown in Figure 11a. The value of internal friction reaches a minimum at 300 °C. By comparison, the internal friction of the C/G/C composite decreases gradually and then remains nearly unchanged past a certain temperature from 350 to 450 °C. The C/C composite possesses a larger overall internal friction than the C/G/C composite.

The internal friction of the material could be expressed as the following Equation (10):

(10) $$Q^{-1}=\frac{∆\omega }{2\pi W}$$

where ∆ω is the energy dissipated per unit volume, and *W* is the maximum stored elastic energy per unit volume.

After assuming only defects of matrix cracks and corresponding interfacial debonding in fibers reinforced composites without broken fibers, the friction work of one load cycle can be estimated by the Equation (11) [51,52]

(11) $$∆\omega =\frac{\pi E_{M}^{2}}{96\upsilon_{M}\tau }{\left[\frac{d(1-\upsilon_{M})∆\sigma }{E_{F}}\right]}^{3}$$

where *d* is the fiber diameter, ∆σ is the fatigue stress range, *E*_F_ is the elastic modulus of fiber, *E_M_* is the elastic modulus of matrix, and τ is the IDS.

The IDS depends on the material and structure of the interface, is the only factor related to the interface. Table 2 shows that the IDS of the composites demonstrate the following order: C/G/C > C/C. Bigger IDS restrains sliding and decreases the friction between the different phases of a composite, which lead to decrease of the energy loss and internal friction, because increase of IDS has a negative dependence on the interfacial sliding stress as far as the sliding actually occurs. Therefore, according to Equation (11), as the IDS decreases, the probability of interfacial sliding and friction increases, and these could induce more energy losses and increase the internal friction of the C/C composites. Some researchers [53,54] reported the similar relationship of the reduction of interfacial shear stress and confirmed internal friction as a critical positive factor in C/C and glass fibers reinforced polyamide composites.

This characteristic indicates that the fiber/matrix interface bonding of the composites exerts significant effects on their properties. In general composite materials, the temperature increase not only reduces or eliminates thermal stresses in the fibers and matrix, leading to enhancements in the bonding of microcracks in the interface and matrix. In addition, the number of some debonded fiber–matrix regions around matrix cracks are reduced, and this phenomenon would increase attrition energy loss [54,55].

By contrast, in the C/G/C composite, graphene sheets are grafted onto the fiber surface, forming a new hierarchical structure with increased carbon fiber surface roughness. As the temperature increases, the internal friction of the C/G/C composite remains nearly unchanged because of improvements in the fiber/matrix interface bonding strength of the composite. This result indicates that graphene significantly affects the evolution of internal friction with temperature.

Dynamic modulus refers to the capability of a material to resist strain under external loading and strongly depends on the integrity and continuity of materials [56]. The temperature dependence of the storage modulus of both composites is shown in Figure 11b. The storage modulus of the C/G/C composite increases slowly with increasing temperature but remains higher than that of the C/C composite, which exhibits a gradual increase and then a slight decrease. When composites are cooled from manufacturing temperature to room temperature, stresses are induced because of mismatches in the thermal expansion coefficients of the carbon matrix and fibers [57]. This mismatch causes debonding and sliding in the interphase and microcracks in the matrix, thereby improving the fiber/matrix interface bonding strength of the composites and enhancing their load transfer abilities.

The mechanical properties of the C/G/C composites in comparison with those of the C/C composites are listed in Table 2. The flexural strength, interlaminar shear strength and storage modulus of the composites increase by 76.4%, 44.6% and 22.8%, respectively, because of the high bonding strength between the carbon fibers and the matrix. Meanwhile, the internal friction of the composites decreases by 42.1% because of a small amount of defects in matrix and interface between fibers and matrix.

### 3.4. Thermal Conductivity

**Figure 12:**
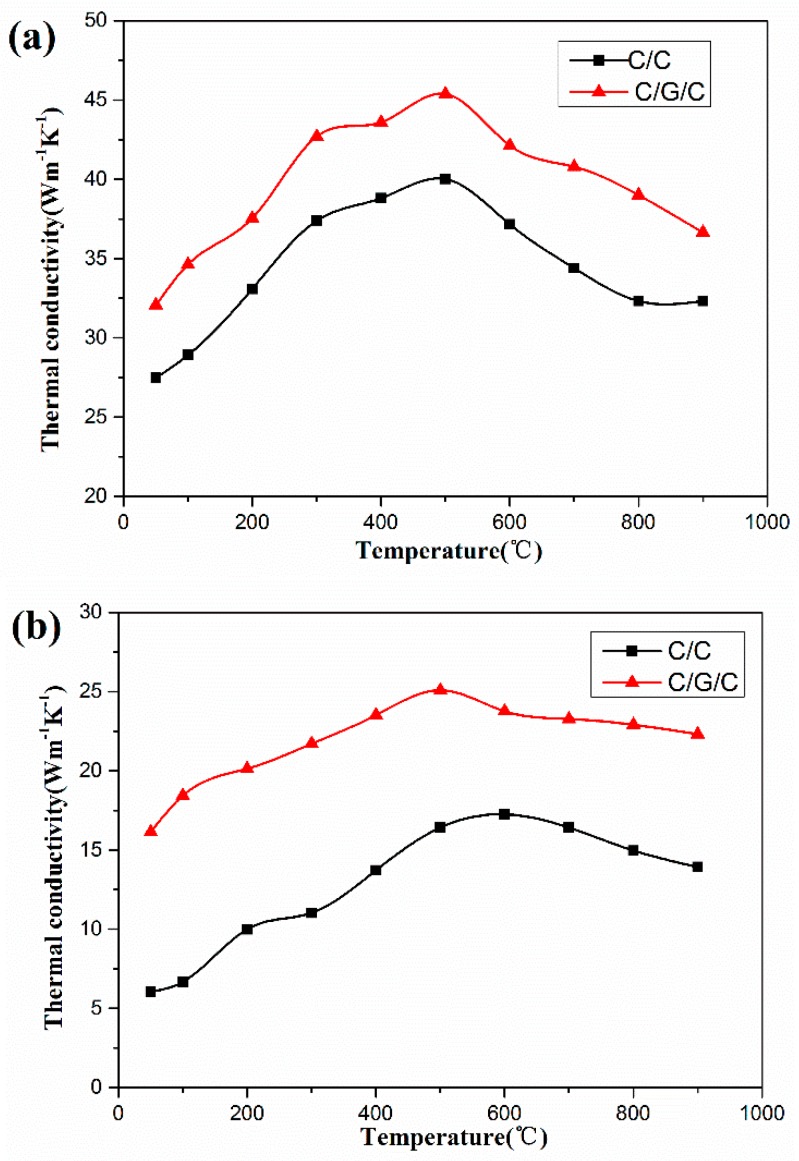
Thermal conductivity of C/C and C/G/C composites in the (**a**) *X*–*Y* and (**b**) *Z* directions.

Figure 12 shows the effect of graphene on thermal conductivities of the composites in the *X*–*Y* and *Z* directions. The thermal conductivities of both composites in the *X*–*Y* and *Z* directions initially increase and then subsequently decrease with increasing temperature. Under the same temperature level, the thermal conductivity of the C/G/C composites is higher than that of C/C composites in both directions at 50–900 °C.

As shown in Figure 12, the thermal conductivities of the C/G/C composite in the *X*–*Y* and *Z* directions are higher than those of the C/C composite by 13.3%–19.8% and 37.7%–176.3%, respectively, at 50–900 °C. This result may be attributed to the easy formation of a percolation channel of graphene sheets on the carbon fibers and the function of graphene sheets as a bridge in the interfacial area and pyrocarbon matrix.

In composites, the thermal conductivity λ is proportional to the outcome of the phonon mean free path, the speed of sound and the heat capacity. Where the phonon contribution to the thermal conductivity dominates in composites [58,59]. And free path is limited by scattering of phonon from defects and boundaries. For graphene, the phonon mean free path is very large, so graphene have extremely high thermal conductivity. Therefore, with graphene modified C/C composites, thermal conductivity of C/G/C composites will get a significant increase.

The results indicate the potential superiority of the C/G/C composite over the C/C composite in terms of thermal conduction. Therefore, research on the thermal properties of the C/G/C composites may help improve the current understanding on the heat transfer way of graphene grafted onto carbon fibers and promote the use of their prominent thermal conduction properties [60].

### 3.5. Frictional Properties

**Figure 13:**
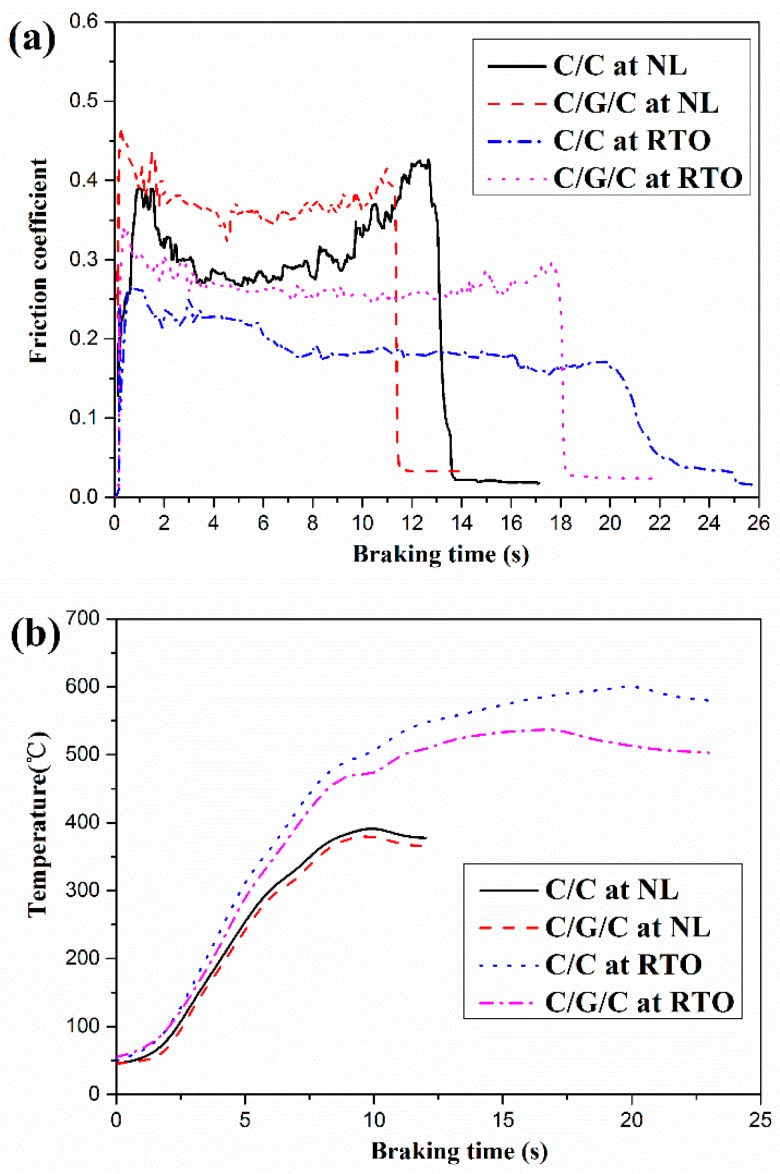
Typical braking curves of the C/C and C/G/C composites at the NL and RTO braking levels: (**a**) Friction coefficient of both composites; (**b**) The surface temperature of both composites.

In the NL and RTO braking condition, the friction coefficient curves of both composites are shown in Figure 13. The average friction coefficients of C/C composites at the NL and RTO braking condition are 0.33 and 0.22, respectively. For C/G/C composites, the average friction coefficients at the NL and RTO braking condition are 0.37 and 0.31, respectively. It is found that the friction coefficient of both composites decreases with increasing braking speed and pressure. This phenomenon is similar to previous results for C/C composites [61,62]. In addition, the stability coefficient of the C/G/C composite is relatively more stable (0.83 at NL; 0.76 at RTO) in comparison with those of the C/C composite (0.61 at NL; 0.47 at RTO). The C/G/C composite exhibited stable friction coefficients than that of previous studies [63,64].

While the temperatures of both composites considerably increase with increasing braking energy, their values are relatively different in Figure 13b. The temperature of the C/G/C composite is always lower than that of the C/C composite, mainly because of the presence of graphene in the former and differences in the structures of their matrices. The maximum temperature of the brake surface is an important factor influencing the performance of braking composites. Extremely high temperatures can cause carbon braking materials to become more susceptible to abrasion or oxidation. Because of the maximum surface temperature of the C/G/C composite (381 °C at NL; 539 °C at RTO) is significantly lower than that of the C/C composites (395 °C at NL; 603 °C at RTO), the former exhibits better thermal diffusion properties than the latter. This finding is consistent with the analysis results of thermal conductivity.

Table 3 reveals that the average friction coefficients of both composites at the NL braking condition are higher than those at the RTO braking condition. As the braking energy increases, the average friction coefficients of the C/G/C composite remain relatively stable, whereas those of the C/C composite present a relatively evident drop from 0.33 to 0.22. The average wear loss of both composites is extremely low at the NL braking level in comparison with that at the RTO level. At both braking levels, the mass wear loss of the C/G/C composite is lower than that of the C/C composite. The friction film always contributes to the stable friction and low wear during the braking. The presence of graphene has played a significant role on the formation of the friction film. In addition, C/G/C composites have strong fiber/matrix interface and low hardness matrix, confirming from its high mechanical strengths of the composites, high texture degree of carbon matrix and self-lubrication of graphene. Thus, we can conclude that the different braking performances of the composites may be attributed to the effect of graphene. These findings demonstrate that graphene play an important role in the frictional properties of the final composite.

**Figure 14:**
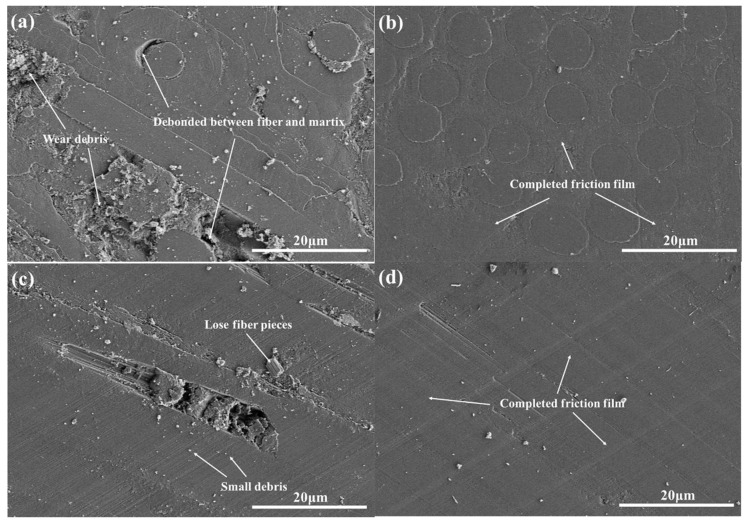
SEM morphologies of the worn surfaces of both composites: (**a**) C/C NL; (**b**) C/G/C NL; (**c**) C/C RTO; and (**d**) C/G/C RTO.

In the NL and RTO braking condition, SEM morphologies of the worn surfaces of both composites are shown in Figure 14. At the NL braking condition, the worn surfaces of the C/C composite is covered by the incomplete friction film and partially appeared wear debris on the worn surface. Owing to the weak fiber/matrix interface bonding strength of C/C composites, several defects and cracks are appeared, such as debonding between the fiber and matrix. These phenomena have negative effects on the formation of the completed friction film. The worn surfaces of the C/G/C composites are covered by a complete friction film because graphene induces improvements in fiber/matrix interface bonding strength and the hardness of matrix deformability. Good mechanical properties are able to maintain for integrality of the worn surfaces [65]. Thus, the wear debris is easier to deform a completed friction film. At the RTO braking level, small debris, loose fiber pieces, and a discontinuous friction film are formed on the surface of the C/C composite. Small broken fiber pieces may induce rolling friction and scratch the friction film away. Rolling wear may explain why the friction coefficient of the C/C composite in the NL test is considerably lower than that in the RTO test. A smooth, uniform, and compact film is formed on the surface of the C/G/C composites and partially filled with small debris. The wear debris of the C/G/C composite is compacted to form a lubricating film that stabilizes the friction coefficient. Owing to the excellent mechanical properties and self-lubricating properties of grapheme [7,8,9,10], a high-strength lubricating film could be easily formed on the surface of the composite, resulting in minimal wear loss. During RTO braking, graphene around the carbon debris forms a lubricating film with high strength, high modulus, and good thermal conductivity with increasing braking energy. The favorable mechanical properties of C/G/C composites observed further contribute to the smoothness of the friction surface. Therefore, the larger friction coefficient of this material in comparison with that of the C/C composite is related to the presence of graphene and pyrocarbon debris, which promote the formation of a friction film. Therefore, the better friction property of the C/G/C composites than that of the C/C composites is related to the fact that the existence of these graphene, better mechanical properties and lower hardness of carbon matrix may promote the formed friction film.

## 4. Conclusions

A simple and effective strategy is developed to prepare C/G/C composites. It demonstrates that microstructure, mechanical, thermal and frictional properties of C/G/C composites are strongly influenced by fiber/matrix interface modified by graphene. PLM, SEM and Raman spectra show that graphene grafted on fiber can modify the pyrocarbon matrix by strongly affecting the deposition behavior of pyrolytic carbon during CVI. Mechanical tests verify that graphene sheets in fiber/matrix interface are more beneficial to obtain higher flexural strength, interlaminar shear strength, and storage modulus in C/G/C composite than that of C/C composites without graphene. Owing to the self-lubricating properties of graphene, the highly textured matrix of the modified composite shows lower hardness and good fiber/matrix interface bonding strength in comparison with those of the unmodified composite. A complete friction film could be easily formed on the friction surface of the modified composite, resulting in stable friction coefficients and lower wear losses at both braking levels tested. According to the surface temperature of both composites at the NL and RTO braking levels, the C/G/C composite exhibits better thermal diffusion properties than the C/C composite. This result is consistent with our analysis results of thermal conductivity. This new method of fabricating C/G/C composites proposed in this work offers new possibilities in the field of aviation materials.

## Figures and Tables

**Table 1:** Friction testing conditions.

Testing Condition	Braking Speed (m/s)	Inertia (kg·m^2^)	Braking Pressure (MPa)
Normal Landing (NL)	25	0.3	0.65
Rejected Take-Off (RTO)	28	0.5	1.25

**Table 2:** Static mechanical performance results and dynamic mechanical properties of the composites at 50 °C, 10 Hz, and 0.025% strain.

Properties	Composite C/C	Composite C/G/C
Flexural strength (MPa)	106 ± 10	187 ± 12
σ_0_ (MPa)	110	188
m *	17.4	19.7
Internal friction	0.038	0.022
Storage modulus (GPa)	12.7	15.6
Interlaminar shear strength (MPa)	10.3 ± 1.5	14.9 ± 1.8
Interfacial debonding strength (MPa)	7.6 ± 1.6	20.4 ± 2.3

*: m represents the slope of the fitted straight line.

**Table 3:** Results of braking experiments at the NL and RTO braking levels.

Performance	Braking Levels	C/C Composites	C/G/C Composites
Friction coefficient	NL	0.33	0.37
	RTO	0.22	0.31
Stability coefficient	NL	0.61	0.83
	RTO	0.47	0.76
Braking energy (J·cm^−2^)	NL	3210	3233
	RTO	5237	5254
Maximum temperature (°C)	NL	395 ± 24	381 ± 23
	RTO	603 ± 31	539 ± 29
Mass wear loss (mg·side^−1^·stop^−1^)	NL	5.84	4.96
	RTO	112.54	98.72

## References

[1] Ozcan S., Tezcan J., Filip P. (2009). Microstructure and elastic properties of individual components of C/C composites. Carbon.

[2] Ozcan S., Tezcan J., Gurung B., Filip P. (2011). The effect of heat treatment temperature on the interfacial shear strength of C/C composites. J. Mater. Sci..

[3] Kasem H., Bonnamy S., Berthier Y., Dufrénocy P., Jacquemard P. (2009). Tribological, physicochemical and thermal study of the abraupt friction transition during carbon/carbon composites friction. Wear.

[4] Vignoles G.L., Aspa Y., Quintard M. (2010). Modelling of carbon-carbon composite ablation in rocket nozzles. Compos. Sci. Technol..

[5] Lee K.J., Cheng H.Z., Chen J.S. (2006). Effect of densification cycles on continuous friction behavior of carbon-carbon composites. Wear.

[6] Blanco C., Bermejo J., Marsh H., Menendez R. (1997). Chemical and physical properties of carbon as related to brake performance. Wear.

[7] Geim A.K. (2009). Graphene: Status and prospects. Science.

[8] Park S., Ruoff R.S. (2009). Chemical methods for the production of graphenes. Nat. Nanotechnol..

[9] Lee C., Wei X.D., Kysar J.W., Hon J. (2008). Measurement of the elastic properties and intrinsic strength of monolayer graphene. Science.

[10] Eswaraiah V., Balasubramaniam K., Ramaprabhu S. (2012). One-pot synthesis of conducting graphene-polymer composites and their strain sensing application. Nanoscale.

[11] Lu X.F., Xiao P. (2016). Effect of carbon nanofiber modification on the tribological properties of C/C composites. New Carbon Mater..

[12] Li G., Yan Q. (2015). Comparison of Friction and Wear Behavior between C/C, C/C-SiC and Metallic Composite Materials. Tribol. Lett..

[13] Mao Z.L., Yang X.J., Zhu S.L., Cui Z.D., Lu Y. (2014). Pack cementation processing parameters for SiC coatings on C/C for optimum tribological properties. Surface Coatings Technol..

[14] Zhao Y.H., Wu Z.K., Bai S.L. (2015). Study on thermal properties of graphene foam/graphene sheets filled polymer composites. Compos. Part A.

[15] Meng Y., Su F.H., Chen Y.Z. (2015). Synthesis of nano-Cu/graphene oxide composites by supercritical CO_2_-assisted deposition as a novel material for reducing friction and wear. Chem. Eng. J..

[16] Mittal G., Dhand V., Rhee K.Y., Park S.J., Lee W.R. (2015). A review on carbon nanotubes and graphene as fillers in reinforced polymer nanocomposites. J. Ind. Eng. Chem..

[17] Manocha L.M., Bhatt H., Manocha S.M. (1996). Development of carbon/carbon composites by co-carbonization of phenolic resin and oxidized PAN fibers. Carbon.

[18] Ko T.H., Kuo W.S., Chang Y.H. (2003). Influence of carbon–fiber felts on the development of carbon-carbon composites. Compos. Part A.

[19] Stankovich S., Piner R.D., Nguyen S.T., Ruoff R.S. (2006). Synthesis and exfoliation of isocyanate-treated graphene oxide nanoplatelets. Carbon.

[20] Stankovich S., Piner R.D., Chen X.Q., Wu N.Q., Nguyen S.T., Ruoff R.S. (2006). Stable aqueous dispersions of graphitic nanoplatelets via the reduction of exfoliated graphite oxide in the presence of poly(sodium 4-styrenesulfonate). J. Mater. Chem..

[21] Hao M.Y., Luo R.Y., Hou Z.H., Yang W., Zhang Y., Yang C.L. (2014). Effect of structure of pyrocarbon on the static and dynamic mechanical properties of carbon/carbon composites. Mater. Sci. Eng. A.

[22] ASTM International (2010). Standard Test Method for Measuring Vibration-Damping Properties of Materials.

[23] Wu X.W., Luo R.Y. (2011). Mechanical properties investigation of carbon/carbon composites fabricated by a fast densification process. Mater. Des..

[24] Aviation Industry Standard (2004). Test Methods for Aircraft Wheel Friction Materials.

[25] ASTM International (2015). Development of Specifications for Fiber Reinforced Carbon-Carbon Composite Structures for Nuclear Applications.

[26] Ozcan S., Filip P. (2005). Microstructure and wear mechanisms in C/C composites. Wear.

[27] Pascal D., Jacques L., Carpentier L., Loubet J.L., Kapsa P. (2002). Sharp indentation behavior of carbon/carbon composites and varieties of carbon. Carbon.

[28] Yang W., Kohyama A., Noda T., Katoh Y., Hinoki T., Araki H., Yu J. (2002). Interfacial characterization of CVI–SiC/SiC composites. J. Nucl. Mater..

[29] Zhao F., Huang Y.D., Liu L., Bai Y.P., Xu L.W. (2011). Formation of a carbon fiber/polyhedral oligomeric silsesquioxane/carbon nanotube hybrid reinforcement and its effect on the interfacial properties of carbon fiber/epoxy composites. Carbon.

[30] Tzounis L.D.S., Rooj S., Fischer D., Mäder E., Das A., Stamm M. (2014). High performance natural rubber composites with a hierarchical reinforcement structure of carbon nanotube modified natural fibers. Mater. Des..

[31] Zhao F., Huang Y.D. (2011). Preparation and properties of polyhedral oligomeric silsesquioxane and carbon nanotube grafted carbon fiber hierarchical reinforcing structure. J. Mater. Chem..

[32] Reznik B., Gerthsen D., Hüttinger K.J. (2001). Micro and nanostructure of the carbon matrix of infiltrated carbon fiber felts. Carbon.

[33] Zhang J.C., Luo R.Y., Xiang Q., Yang C.L. (2011). Compressive fracture behavior of 3D needle-punched carbon/carbon composites. Mater. Sci. Eng. A.

[34] Guellali M., Oberacker R., Hoffmann M.J. (2005). Influence of the matrix microstructure on the mechanical properties of CVI-infiltrated carbon fiber felts. Carbon.

[35] Dong G.L., Huttinger K.J. (2002). Consideration of reaction mechanisms leading to pyrolytic carbon of different textures. Carbon.

[36] Thostenson E.T., Ren Z.F., Chou T.W. (2001). Advances in the science and technology of carbon nanotubes and their composites: A review. Compos. Sci. Technol..

[37] Gong Q.M., Li Z., Bai X., Li D., Liang J. (2005). The effect of carbon nanotubes on the microstructure and morphology of pyrolytic carbon matrices of C–C composites obtained by CVI. Compos. Sci. Technol..

[38] Leszek N., Paul W., Jagodzinski P. (1993). Raman spectroscopic characterization of graphites: A reevaluation of spectra/structure correlation. Carbon.

[39] Malard L.M., Pimenta M.A., Dresselhaus G., Dresselhaus M.S. (2009). Raman spectroscopy in graphene. Phys. Rep..

[40] Naslain R.R., Pailler R.J., Lamon J.L. (2010). Single and multilayered interphases in SiC/SiC composites exposed to severe environmental conditions: An overview. Int. J. Appl. Ceram. Technol..

[41] Jiang D.W., Liu L., Long J., Xing L.X., Huang Y.D., Wu Z.J. (2014). Reinforced unsaturated polyester composites by chemically grafting amino-POSS onto carbon fibers with active double spiral structural spiral phosphodicholor. Compos. Sci. Technol..

[42] Liu X.Y., Song Y., Li C.M., Wang F.P. (2012). Synthesis and characterization of carbon fibers reinforcement with grafted graphene oxide. Chin. J. Inorg. Chem..

[43] Zhang X.Q., Fan X.Y., Yan C., Li H.Z., Zhu Y.D., Li X.T. (2012). Interfacial microstructure and properties of carbon fiber composites modified with graphene oxide. ACS Appl. Mater. Interfaces.

[44] Mei L., He X.D., Li Y.B., Wang R.G., Wang C., Peng Q.Y. (2010). Grafting carbon nanotubes onto carbon fiber by use of dendrimers. Mater. Lett..

[45] Lin J.H., Huang C.L., Liu C.F., Chen C.K., Lin Z.I., Lou C.W. (2015). Polypropylene/Short Glass Fibers Composites: Effects of coupling agents on mechanical Properties, thermal Behaviors, and morphology. Materials.

[46] Munz D., Fett T. (1999). Ceramics: Mechanical, Properties, Failure Behaviour, Materials Selection, Corrected Edition.

[47] Li J., Luo R.Y., Bi Y.H., Xiang Q., Lin C., Zhang Y.F. (2008). The preparation and performance of short carbon fiber reinforced adhesive for bonding carbon/carbon composites. Carbon.

[48] Chen J., Xiao P., Xiong X. (2015). The mechanical properties and thermal conductivity of carbon/carbon composites with the fiber/matrix interface modified by silicon carbide nanofibers. Mater. Des..

[49] Zhang H., Guo L., Song Q., Fu Q.G., Li H., Li K. (2013). Microstructure and flexural properties of carbon/carbon composite with in-situ grown carbon nanotube as secondary reinforcement. Prog. Nat. Sci..

[50] Li D., Luo G., Yao Q., Jiang N., Jiang L. (2015). High temperature compression properties and failure mechanism of 3D needle-punched carbon/carbon composites. Mater. Sci. Eng. A.

[51] Cho C., Holmes J.W., Barber J.R. (1991). Estimation of interfacial shear in ceramic composites from frictional heating measurements. J. Am. Ceram. Soc..

[52] Cho C., Choi E.Y., Beom H.G., Kim C.B. (2005). Micro-frictional dissipation in fiber-reinforced ceramic matrix composites and interfacial shear estimation with a consideration of uneven fiber packing. J. Mater. Process. Technol..

[53] Yasuo K., Yoshie L., Naohiro I., Ken’ich O. (2003). Internal friction of carbon–carbon composites at elevated temperatures. J. Alloys Compd..

[54] Sato S., Serizawa H., Araki H., Noda T., Kohyama A. (2003). Temperature dependence of internal friction and elastic modulus of SiC/SiC composites. J. Alloys Compd..

[55] Zhang J., Xu Y.D., Zhang L.T., Cheng L.F. (2005). Internal friction and dynamic modulus of three-dimensional silicon carbide-matrix composites. Mater. Lett..

[56] Hou Z.H., Luo R.Y., Yang W., Xu H.Z., Han T. (2016). Effect of fiber directionality on the static and dynamic mechanical properties of 3D SiCf/SiC composites. Mater. Sci. Eng. A.

[57] Qiao S.R., Shaorong Z., Shihong B., Xiaoyan S. (1997). The internal friction of unidirectional CC composites fabricated in a magnetic field. Carbon.

[58] Berber S., Kwon Y.K., Tomanek D. (2000). Unusually high thermal conductivity of carbon nanotubes. Phys. Rev. Lett..

[59] Pop E., Mann D., Wang Q., Goodson K., Dai H.J. (2006). Thermal conductivity of an individual single-wall carbon nanotube above room temperature. Nano Lett..

[60] Yun C., Feng Y.B., Qiu T., Yang J., Li X.Y., Yu L. (2015). Mechanical, electrical, and thermal properties of graphene nanosheet/aluminum nitride composites. Ceram. Int..

[61] Lee K.J., Kuo H.H., Chen J.H., Lin N., Ju C.P. (1999). Effect of surface condition on tribological behavior of PAN-CVI based carbon/carbon composite. Mater. Chem. Phys..

[62] Cai Y.Z., Yin X.W., Fan S.W., Zhang L.T., Cheng L.F. (2013). Tribological behavior of three-dimensional needled ceramic modified carbon/carbon composites in seawater conditions. Compos. Sci. Technol..

[63] Krenkel W., Berndt F. (2005). C/C–SiC composites for space applications and advanced friction systems. Mater. Sci. Eng. A.

[64] Li Z., Xiao P., Xiong X., Zhu S.H. (2008). Tribological characteristics of C/C–SiC braking composites under dry and wet conditions. Trans. Nonferr. Metal. Soc..

[65] Zhang D.Y., Lin P., Dong G.N., Zeng Q.F. (2013). Mechanical and tribological properties of self-lubricating laminated composites with flexible design. Mater. Des..

